# Identification of seven novel ferroptosis-related long non-coding RNA signatures as a diagnostic biomarker for acute myeloid leukemia

**DOI:** 10.1186/s12920-021-01085-9

**Published:** 2021-09-27

**Authors:** Zhiyuan Zheng, Wei Wu, Zehang Lin, Shuhan Liu, Qiaoqian Chen, Xiandong Jiang, Yan Xue, Donghong Lin

**Affiliations:** 1 Medical Technology and Engineering College of Fujian Medical University, Fuzhou, 350001 Fujian China; 2 Medical Technology Experimental Teaching Center of Fujian Medical University, Fuzhou, 350001 Fujian China; 3 School of Computer and Information Engineering, Xiamen University of Technology, Xiamen, 361024 Fujian China

**Keywords:** Ferroptosis, Acute myeloid leukemia, Long non-coding RNA signature, Prognosis, TCGA

## Abstract

**Background:**

Ferroptosis is a newly discovered type of programmed cell death that participates in the biological processes of various cancers. However, the mechanism by which ferroptosis modulates acute myeloid leukemia (AML) remains unclear. This study aimed to investigate the role of ferroptosis-related long non-coding RNAs (lncRNAs) in AML and establish a corresponding prognostic model.

**Methods:**

RNA-sequencing data and clinicopathological characteristics were obtained from The Cancer Genome Atlas database, and ferroptosis-related genes were obtained from the FerrDb database. The “limma” R package, Cox regression, and the least absolute shrinkage and selection operator were used to determine the ferroptosis-related lncRNA signature with the lowest Akaike information criteria (AIC). The risk score of ferroptosis-related lncRNAs was calculated and patients with AML were divided into high- and low-risk groups based on the median risk score. The Kaplan–Meier curve and Cox regression were used to evaluate the prognostic value of the risk score. Finally, gene set enrichment analysis (GSEA) and single-sample gene set enrichment analysis (ssGSEA) were performed to explore the biological functions of the ferroptosis-related lncRNAs.

**Results:**

Seven ferroptosis-related lncRNA signatures were identified in the training group, and Kaplan–Meier and Cox regression analyses confirmed that risk scores were independent prognostic predictors of AML in both the training and validation groups (All *P* < 0.05). In addition, the area under the curve (AUC) analysis confirmed that the signatures had a good predictive ability for the prognosis of AML. GSEA and ssGSEA showed that the seven ferroptosis-related lncRNAs were related to glutathione metabolism and tumor immunity.

**Conclusions:**

In this study, seven novel ferroptosis-related lncRNA signatures (AP001266.2, AC133961.1, AF064858.3, AC007383.2, AC008906.1, AC026771.1, and KIF26B-AS1) were established. These signatures were shown to accurately predict the prognosis of AML, which would provide new insights into strategies for the development of new AML therapies.

**Supplementary Information:**

The online version contains supplementary material available at 10.1186/s12920-021-01085-9.

## Introduction

Acute myeloid leukemia (AML) is a clonal malignant proliferative disease of myeloid blasts of the hematopoietic system. The condition is characterized by impaired differentiation and uncontrolled clonal expansion of myeloid progenitor/precursor cells, leading to bone marrow failure and impaired hematopoietic function [1]. Despite advanced progression in anti-leukemic drug mining [2], the low overall survival (OS) rate has not significantly improved because of the complex tumor microenvironment, evolving dominant clones, and complex molecular mechanisms [3]. Therefore, additional research on useful prognostic biomarkers and possible therapeutic targets is needed to achieve individualized precision therapy.

Ferroptosis is an iron-dependent programmed cell death characterized by the accumulation of intracellular reactive oxygen species [4]. Increasing evidence shows that ferroptosis is involved in various important pathological processes, including cancer development, cardiovascular disease, and ischemia–reperfusion injury [5, 6]. Moreover, research has shown that leukemia cells are more sensitive to the ferroptosis inducer erastin than other cancer cell type [7, 8]. However, the regulation of ferroptosis remains unclear and is far from being used as a treatment strategy for AML. Consequently, identifying the key molecules involved in ferroptosis is a key step in expanding treatment options for patients with AML.

Long non-coding RNAs (lncRNAs) are transcripts of > 200 nucleotides that typically do not encode proteins [9]. lncRNAs play an important role in many life activities, such as epigenetic regulation, cell cycle regulation, and regulation of cell differentiation [10]. In particular, the abnormal function or expression of lncRNA may be closely related to various biological processes, including ferroptosis [11]. For example, the lncRNA LINC00336 inhibits ferroptosis in lung cancer by functioning as a competitive endogenous RNA [12]. The lncRNA P53RRA promotes ferroptosis and apoptosis in cancer via nuclear sequestration of p53 to induce a tumor suppressor effect [13]. Therefore, there are sufficient reasons to determine the ferroptosis-related lncRNAs used to predict the prognosis of patients with AML, which would provide a theoretical basis for the precise treatment of patients with AML.

In the present study, we downloaded gene expression data of AML cohorts from The Cancer Genome Atlas (TCGA) database and identified ferroptosis-related lncRNA signatures that are associated with the prognosis of AML. Because the immune microenvironment is closely related to ferroptosis and the prognosis of patients with AML [14, 15], we further explored the relationship between this signature and immune infiltration. Overall, the ferroptosis-related lncRNA signature might facilitate the effective and accurate prediction of the prognosis of patients with AML and provide new insights into the mechanisms of ferroptosis and developing novel immunotherapies.

## Materials and methods

### Data collection

Gene expression profiles and clinical data from patients with AML were downloaded from TCGA database (https://portal.gdc.cancer.gov/). Annotation of lnc RNAs was obtained from the GENCODE website (https://www.gencodegenes.org/). We collected clinical data including sex; age; French-American-British [FAB] subtype; cytogenetic risk category; and FMS-like tyrosine kinase 3 [FLT3], isocitrate dehydrogenase 1 [IDH1] R132, IDH1 R140, and cytoplasmic nucleophosmin [NPMc] mutations; and follow-up time. We initially obtained 151 samples of TCGA-LAML, and then we screened the samples according to the following criteria: 1) samples were obtained from bone marrow of patients diagnosed with AML according to relevant diagnostic criteria, 2) samples included complete clinical survival information, and 3) the patient's survival time is greater than 30 days. Finally, we included data from 130 patients and randomly assigned them to the training (*n* = 92) and validation (*n* = 38) groups using the caret package in R.

### Ferroptosis-associated lncRNAs

We obtained 259 ferroptosis-associated gene sets from FerrDb (http://www.zhounan.org/ferrdb), the first manually curated resource for regulators and markers of ferroptosis, which was released in January 2020 [16]. To obtain high-quality lncRNA, we excluded those with a low expression level using a cut-off criterion for the average expression in all samples of > 0.5. We then used the lima package in R to analyze the correlation between the expression levels of ferroptosis-related genes and lncRNA in AML samples, and set the threshold conditions to a correlation coefficient > 0.4 and a *P* < 0.001 to detect ferroptosis-associated lncRNAs.

### Establishment of prognostic ferroptosis-associated lncRNA signature

Univariate Cox regression analysis was used to screen for lncRNAs associated with OS in patients with AML. Further screening was based on the least absolute shrinkage and selection operator (LASSO) regression to avoid overfitting. Through the “glmnet package” of the R language [17], we adjusted the L1 penalty parameter using a tenfold cross-validation to reduce the number of genes. Genes with a repetition frequency > 900 times in 1000 replacement samples were considered to be more closely related to OS than those with a lower frequency. Next, to establish a prognostic lncRNA signature, we performed a multivariate Cox regression analysis and then constructed an lncRNA signature with the lowest Akaike information criteria (AIC) value [18]. The risk score for each patient with AML was calculated based on the lncRNA expression level and the corresponding coefficient using the following equation:

$$\begin{aligned} & \beta {\text{lncRNA1}} \times {\text{Expression\;lncRNA1}} + \beta {\text{lncRNA2}} \\ & \quad \times {\text{Expression\;lncRNA2}} + \cdots + \beta {\text{lncRNAn}} \times {\text{Expression\;lncRNAn}}. \\ \end{aligned}$$

### Evaluation of risk score prediction ability

Patients in the training and validation groups were divided into high- and low-risk groups using the median risk score of the training group. The Kaplan–Meier method was used to draw the survival curve. The prediction performance was evaluated using a receiver operating characteristic (ROC) curve. Subsequently, we performed principal component analysis (PCA) [19] and *t*-distributed stochastic neighbor embedding (*t*-SNE) [20] to reduce the dimensions and visualize different ferroptosis statuses based on high- and low-risk groups.

### Construction of ferroptosis-related lncRNA‐mRNA co‐expression network

The correlation between ferroptosis-related lncRNAs and their co‐expressed ferroptosis-related-mRNAs was analyzed using a co‐expression network and Sankey diagram. Cytoscape software (version 3.8.2, http://www.cytoscape.org/) and ggalluvial R package were used to visualize the co-expression network and Sankey diagram [21].

### Gene set enrichment analysis (GSEA)

The potential biological functions of the high- and low‐risk group patients were comparatively investigated using a gene set enrichment analysis (GSEA, version 4.1.0 http://www.gsea-msigdb.org/gsea/index.jsp), and a normal *P* < 0.05 and false discovery rate (FDR, q‐value) < 0.10, were regarded as statistically significant [22].

### Estimation of immune infiltration status

To further explore the relationship between ferroptosis status and immune cell infiltration, We used single-sample gene set enrichment analysis (ssGSEA), and calculated the infiltration scores of 16 immune cells and 13 immune-related pathways [23] using the "gsva package" [24] in R software. In short, based on the expression matrix of a set of genes corresponding to specific immune cells and immune-related pathways, the enrichment scores of different patients are calculated through the ssGSEA algorithm followed by normalization. Finally, the enrichment scores of immune cells and pathways in patients with different risk scores were analyzed to illustrate the potential correlation between ferroptosis and immune infiltration status. The related reference gene file is supplied in Additional file 1.

### Statistical analysis

Chi-square tests were used to compare clinicopathological characteristics between the training and validation groups. Pearson’s correlation test was used to analyze the correlations. The Mann–Whitney U test was used to compare the relationship between clinicopathological characteristics and lncRNA expression. All statistical analyses were performed using R (version 4.0.1, www.r-project.org) and the statistical significance was set at *P* < 0.05.

## Results

### Acquisition of ferroptosis-related lncRNA

**Fig. 1:**
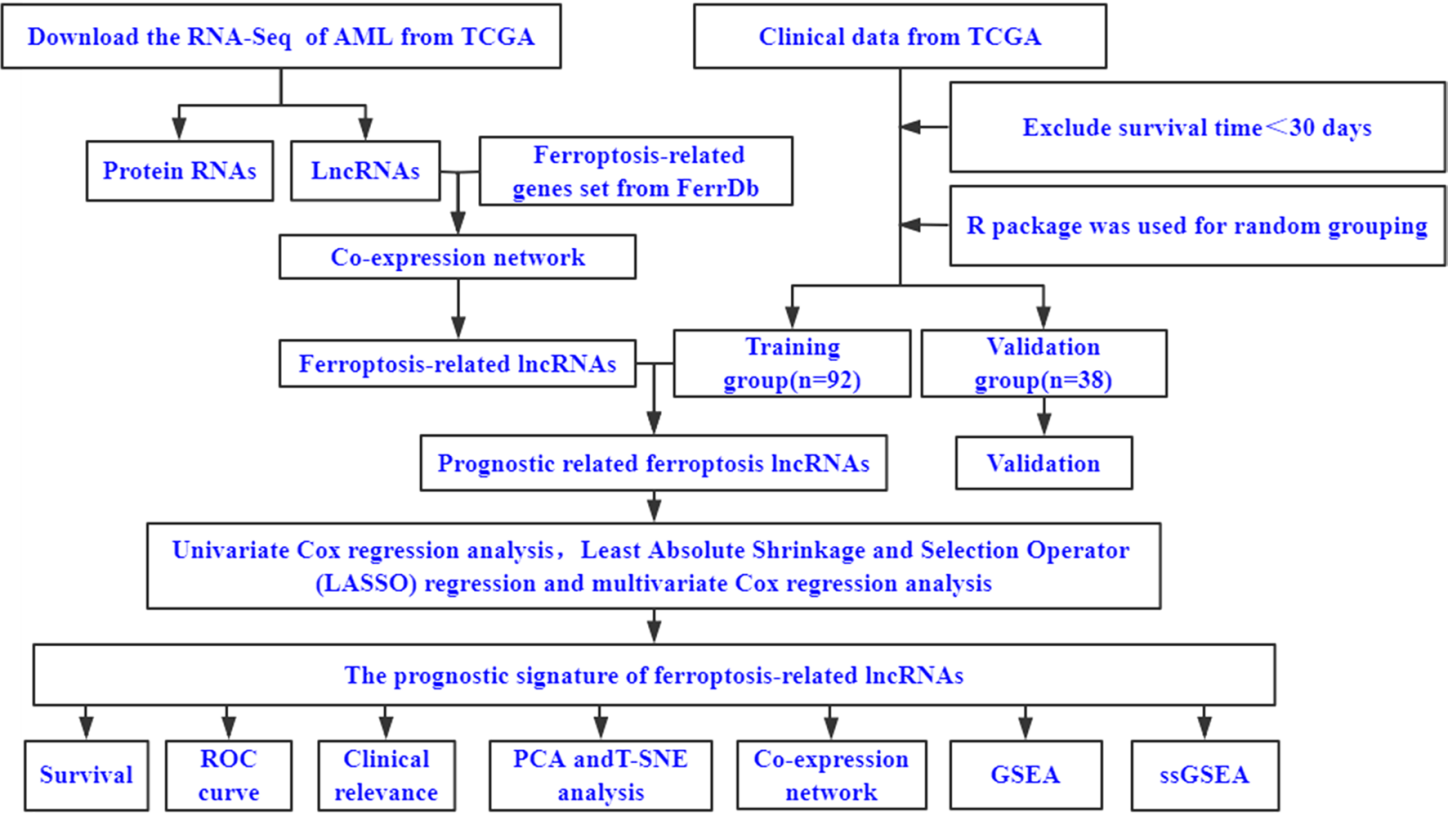
Study flow chart. AML, Acute myeloid leukemia; TCGA, The Cancer Genome Atlas database; LncRNAs, Long non-coding RNAs; t-SNE, *t*-distributed stochastic neighbor embedding; PCA, principal component analysis; GSEA, gene set enrichment analysis; ssGSEA, single-sample gene set enrichment analysis

Figure 1 presents a flow diagram of this study, and 259 ferroptosis-related genes were retrieved from the FerrDb (Additional file 2). We obtained 14,128 lncRNAs from TCGA-LAML, and then used the “limma package” in R studio to obtained 2977 ferroptosis-related lncRNAs. The clinicopathological characteristics of the 130 patients with AML are detailed in Table 1 and the group comparison showed no difference between the training and validation groups.

**Table 1:** Characteristics of patients with acute myeloid leukemia (AML)

Characteristics	Patients (*N*,%)	(*N*,%)		*P*
		Training group (*n* = 92)	Validation group (*n* = 38)	
Sex				0.948
Female	61 (46.9)	43 (46.7)	18 (47.4)	
Male	69 (53.1)	49 (53.3)	20 (52.6)	
Age,years				0.123
≤ 55	65 (50.0)	50 (54.3)	15 (39.5)	
> 55	65 (50.0)	42 (45.7)	23 (60.5)	
FAB subtype				0.706
M0	12 (9.2)	11 (12.0)	1 (2.6)	
M1	31 (23.8)	20 (21.7)	11 (28.9)	
M2	32 (24.6)	21 (22.8)	11 (28.9)	
M3	13 (10.0)	10 (10.8)	3 (7.9)	
M4	27 (20.8)	19 (20.7)	8 (21.1)	
M5	12 (9.2)	8 (8.7)	4 (10.6)	
M6	2 (1.6)	2 (2.2)	0 (0)	
M7	1 (0.8)	1 (1.1)	0 (0)	
Cytogenetics risk category				
Favorable	29 (22.3)	24 (26.1)	5 (13.2)	0.349
Intermediate/Normal	72 (55.4)	50 (54.3)	22 (57.9)	
Poor	27 (20.8)	17 (18.5)	10 (26.3)	
Unknow	2 (1.5)	1 (1.1)	1 (2.6)	
FLT3 mutation				0.937
Positive	33 (25.4)	24 (26.1)	9 (23.7)	
Negative	93 (71.5)	65 (70.6)	28 (73.7)	
Unknow	4 (3.1)	3 (3.3)	1 (2.6)	
IDH1 R132				0.704
Positive	13 (10.0)	10 (10.9)	3 (7.9)	
Negative	116 (89.2)	81 (88.0)	35 (92.1)	
Unknow	1 (0.8)	1 (1.1)	0 (0)	
IDH1 R140				0.337
Positive	11 (8.5)	6 (6.5)	5 (13.5)	
Negative	116 (89.9)	85 (92.4)	31 (83.8)	
Unknow	2 (1.6)	1 (1.1)	1 (2.7)	
NPMc				0.811
Positive	31 (23.8)	22 (23.9)	9 (23.7)	
Negative	98 (75.4)	69 (75.0)	29 (76.3)	
Unknow	1 (0.8)	1 (1.1)	0 (0)	
Follow-up state				0.123
Alive	51 (39.2)	40 (43.5)	11 (28.9)	
Died	79 (60.8)	52 (56.5)	27 (71.1)	

*FAB* French–American–British, *FLT3* FMS-like tyrosine kinase 3, *IDH1* isocitrate dehydrogenase 1, *NPMc* nucleophosmin mutations

### Development and validation of prognostic ferroptosis-related lncRNA signatures

**Fig. 2:**
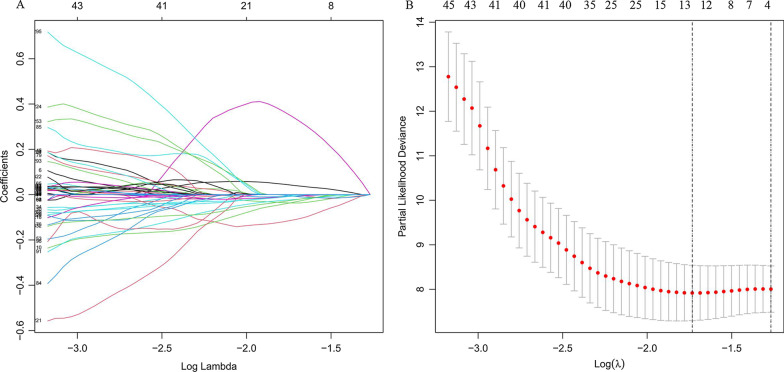
Development of prognostic ferroptosis-related long non-coding RNA (lncRNA) signature. **a**, **b** LASSO Cox regression was performed to identify ferroptosis-related lncRNAs closely associated with prognosis of acute myeloid leukemia (AML). LASSO, Least Absolute Shrinkage and Selection Operator

We constructed a ferroptosis-related lncRNA signature based on the training groups. Furthermore, we performed a preliminary screening using a univariate Cox analysis and obtained 344 ferroptosis-related lncRNAs associated with OS (*P* < 0.05, Additional file 3). Based on the LASSO regression analysis subsequently performed for these genes using a tenfold cross-validation, we screened 13 ferroptosis-related lncRNAs with a repetition rate > 900 times in 1000 replacement samples (Fig. 2A and B). Finally, we performed a multivariate Cox regression analysis and then constructed an lncRNA signature with the lowest AIC value, generating 7 ferroptosis-related lncRNA signatures to predict the prognosis (Table 2). The median risk score classified patients into high-risk (*n* = 46) or low-risk (*n* = 46) groups, which was calculated as follows:

**Table 2:** Seven ferroptosis-related long non-coding RNAs (lncRNAs) detected using multivariable Cox regression analysis

ID	Coef	HR	HR.95L	HR.95H	*P* value
AP001266.2	− 0.1258	0.8817	0.7837	0.9920	0.03642366
AC133961.1	0.7448	2.1060	1.2092	3.6678	0.00850935
AF064858.3	0.1299	1.1387	1.0343	1.2537	0.00809829
AC007383.2	− 0.0913	0.9127	0.8355	0.9971	0.04287405
AC008906.1	− 0.2202	0.8023	0.6821	0.9437	0.00781153
AC026771.1	− 0.1015	0.9035	0.8249	0.9895	0.02875161
KIF26B-AS1	− 0.3423	0.7102	0.5314	0.9490	0.02066836

*coef* coefficient, *HR* hazard ratio

Risk score = −0.1258 × AP001266.2 Expression + 0.7448 × AC133961.1 Expression + 0.1299 × AF064858.3 Expression − 0.0913 × AC007383.2 Expression − 0.2202 × AC008906.1 Expression − 0.1015 × AC026771.1 Expression − 0.3423 × KIF26B-AS1 Expression.

**Fig. 3:**
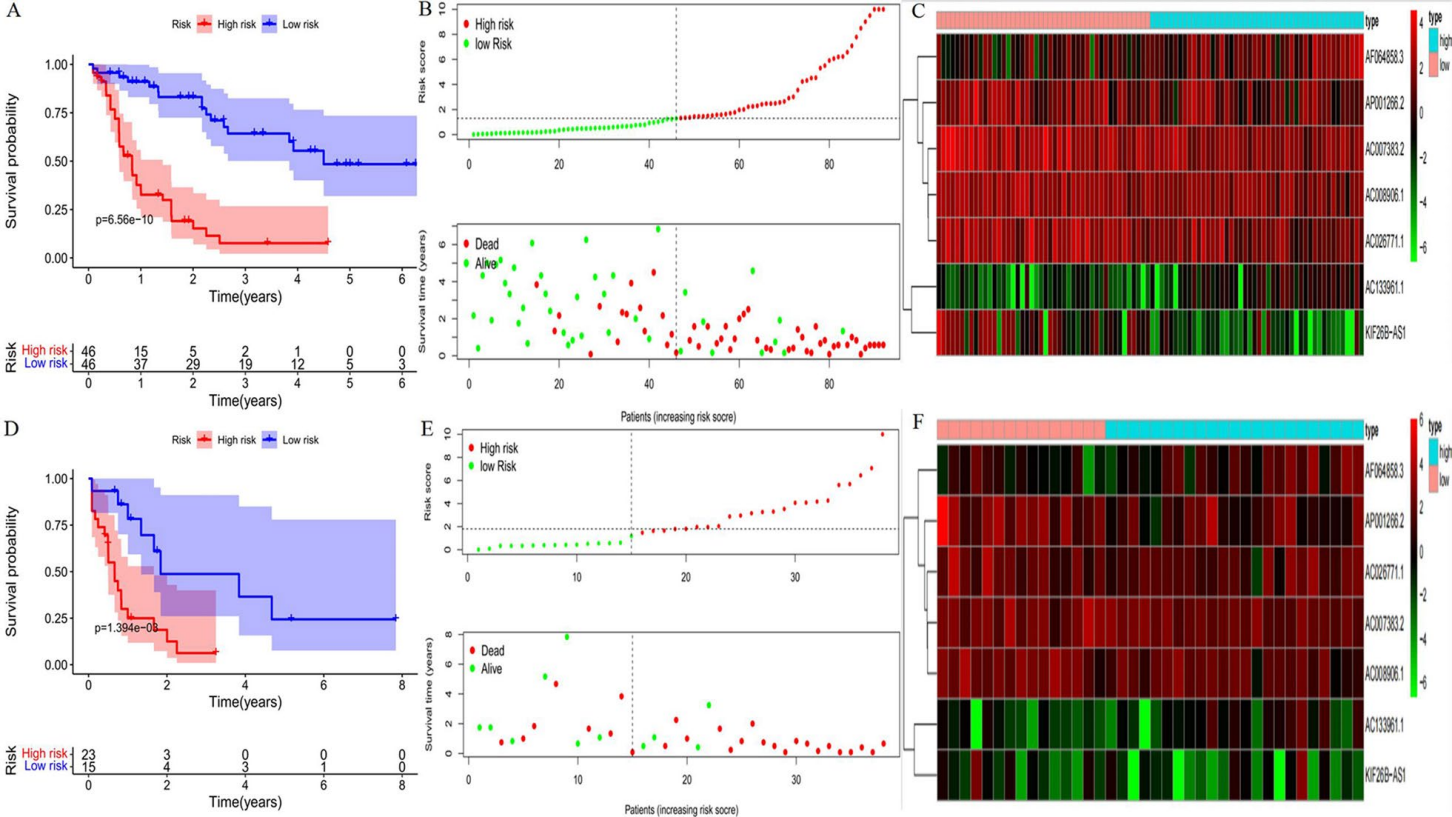
Development and validation of prognostic ferroptosis-related long non-coding RNA (lncRNA) signature. **a** Kaplan–Meier curve, **b** risk score and survival status, and **c** heatmap for training group. **d** Kaplan–Meier curve, **e** risk score and survival status, and (F) heatmap for validation group

The Kaplan–Meier curve showed that patients in the training group with high-risk scores had a significantly higher probability of death than those with low-risk scores (*P* < 0.001, Fig. 3a). The risk of death of the patients increased with increasing risk score, and the survival time decreased (Fig. 3b). The risk heat map clearly shows the expression of different lncRNAs in the high- and low-risk groups (Fig. 3c). Next, we validated our results in a validation group.

**Fig. 4:**
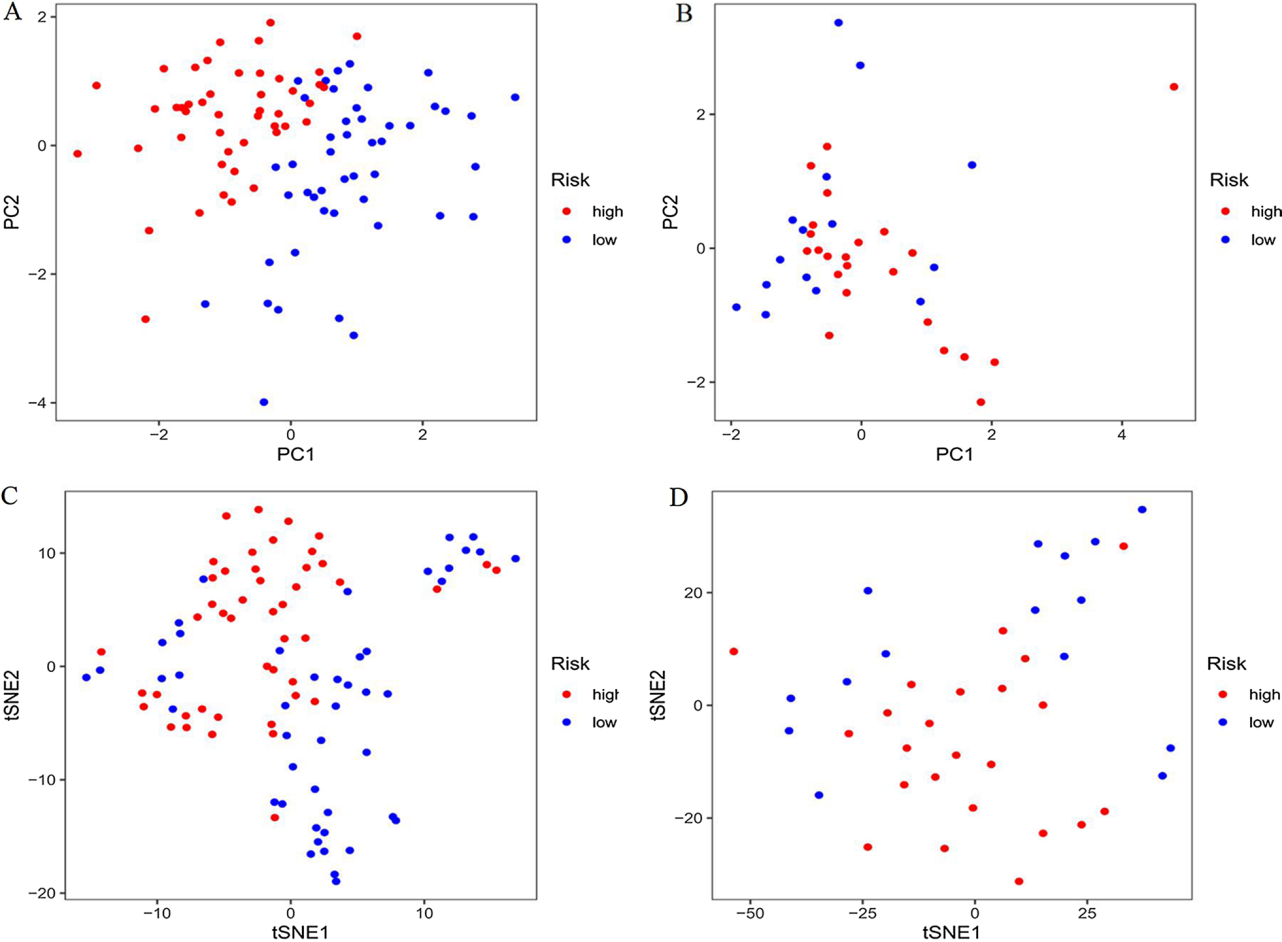
**a**, **b** Principal component analysis (PCA) was performed in training group and validation group, respectively. **c**, **d** *t*-Distributed stochastic neighbor embedding (t-SNE) analysis was performed in the training and validation groups, respectively

We also observed significant differences in OS between the high- and low-risk groups (*P* < 0.01, Fig. 3d, e), and the corresponding gene expression is shown in the heat map (Fig. 3f), which confirmed the accuracy of our results. We further used the Mann–Whitney *U* test to explore the difference in lncRNA expression between the high- and low-risk groups in the entire corhort including all patients (Additional file 4: Fig. S1). The results showed that the expression of lncRNA including AP001266.2, AC133961.1, AF064858.3, AC007383.2, AC008906.1, AC026771.1, and KIF26B-AS1 were significantly different in the entire corhort. We explored the distribution of the high- and low-risk groups using PCA (Fig. 4a, b) and t-SNE (Fig. 4c, d) and intuitively perceived that patients with AML could be better distinguished based on prognosis indicated by lncRNAs associated with ferroptosis.

### Independent prognostic analysis of OS

**Fig. 5:**
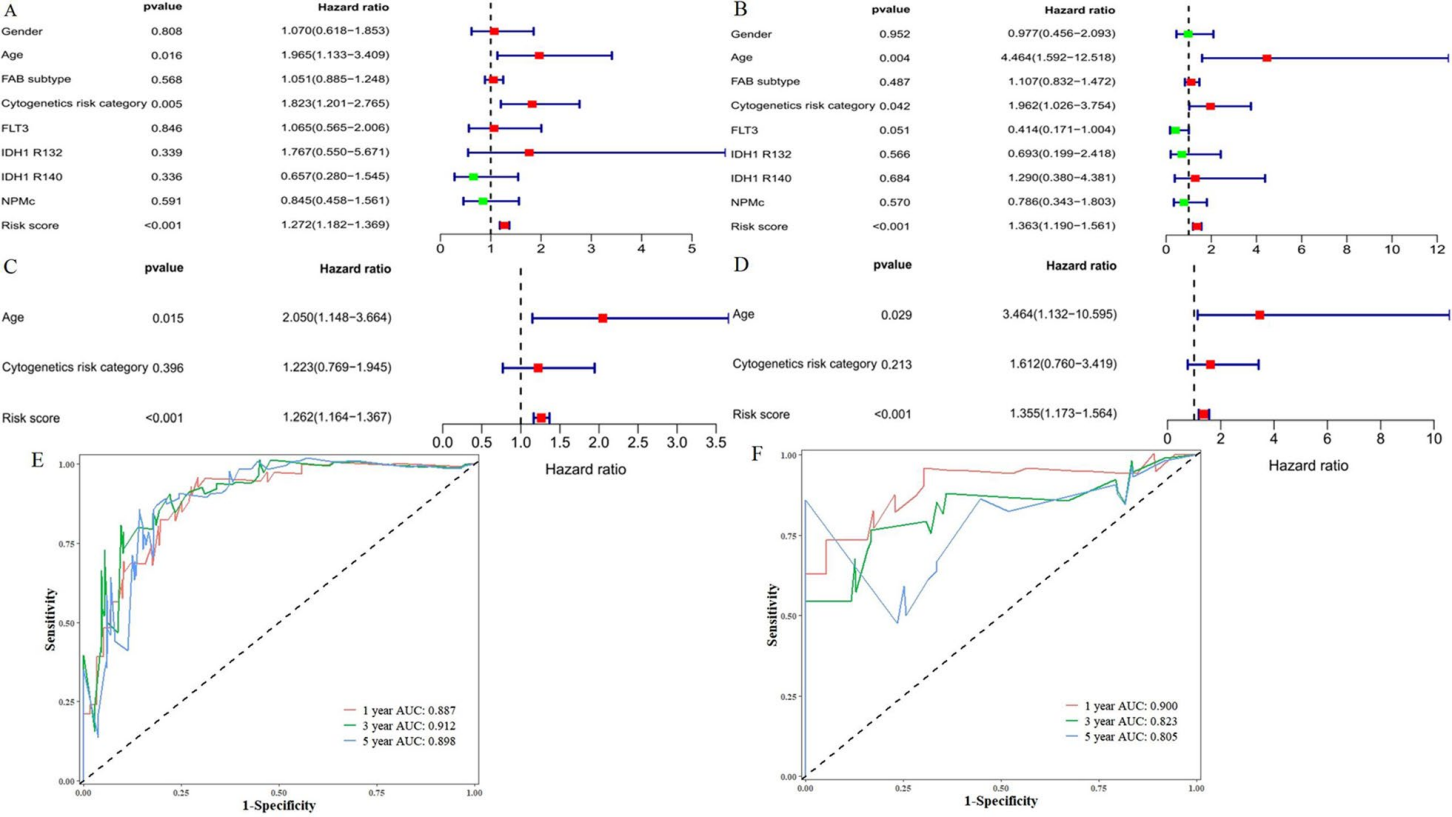
Independent prognostic factors for acute myeloid leukemia (AML) overall survival (OS). Univariate Cox regression analysis in **a** training and **b** validation sets. Multivariate Cox regression analysis in **c** training and **d** validation sets with receiver operating characteristic curve (ROC) analysis of risk scores based on 1-, 3-, and 5-year OS in **e** training and **f** validation sets

Univariate and multivariate Cox regression analyses were performed to assess whether clinical characteristics (sex; age; FAB subtype; cytogenetic risk category; FLT3, IDH1 R132, IDH1 R140, and NPMc mutations) and risk score were independent prognostic factors for OS. In both the training and validation groups, we found that age and risk score were independent prognostic predictors of OS in the univariate and multivariate Cox regression analyses (Fig. 5a–d). In addition, in the time-dependent ROC curve analysis, the area under the curve (AUC) of the OS for 1, 3, and 5 years was 0.887, 0.912, and 0.898 in the training group (Fig. 5e) and 0.900, 0.823, and 0.805 (Fig. 5f) in the validation group, respectively, which further confirms the validity of our results.

### Correlation between expression level of 7 ferroptosis-related lncRNAs and clinicopathological characteristics

**Fig. 6:**
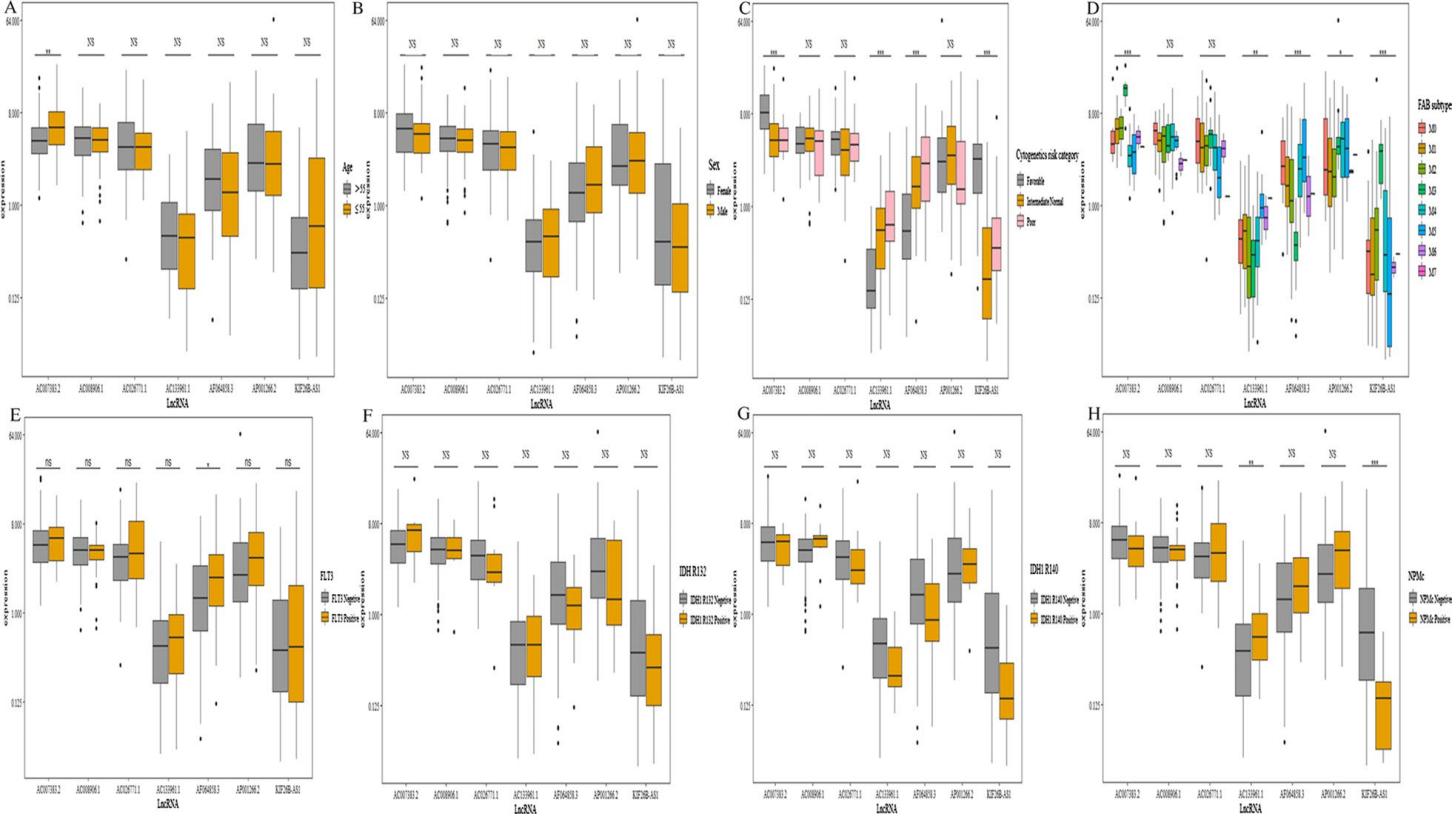
Correlation between expression level of seven ferroptosis-related long non-coding RNAs (lncRNAs) and clinicopathological characteristics in entire corhort. **a**–**h** Age, sex, cytogenetics risk category, French-American-British (FAB) subtype, FMS-like tyrosine kinase 3 (FLT3) mutation, isocitrate dehydrogenase 1 (IDH1) R132, IDH1 R140, and cytoplasmic nucleophosmin (NPMc), respectively. NS: not significant; *P < 0.05; **P < 0.01; ***P < 0.001

We further explored the relationship between clinicopathological characteristics and expression of the lncRNA in the entire corhort. AC007383.2, which was found to decrease with age (Fig. 6a). The high expression levels of AC133961.1 and AF064858.3 were associated with poor risks categorized by cytogenetics, whereas AC007383.2 and KIF26B-AS1 showed an opposite association (Fig. 6c). The expression levels of AC007383.2, AC133961.1, AF064858.3, AP001266.2, and KIF26B-AS1 were related to the FAB subtype (Fig. 6d). The expression of AF064858.8 was correlated with FLT3 mutation (Fig. 6e), and that of AC133961.1 and KIF26B-AS1 was correlated with NPMC mutation (Fig. 6h). However, for sex, IDH1 R132, and IDH1 R140, no statistical difference was observed in the expression levels of each lncRNA (Fig. 6b, f, g).

### Construction of co-expression network and GSEA

**Fig. 7:**
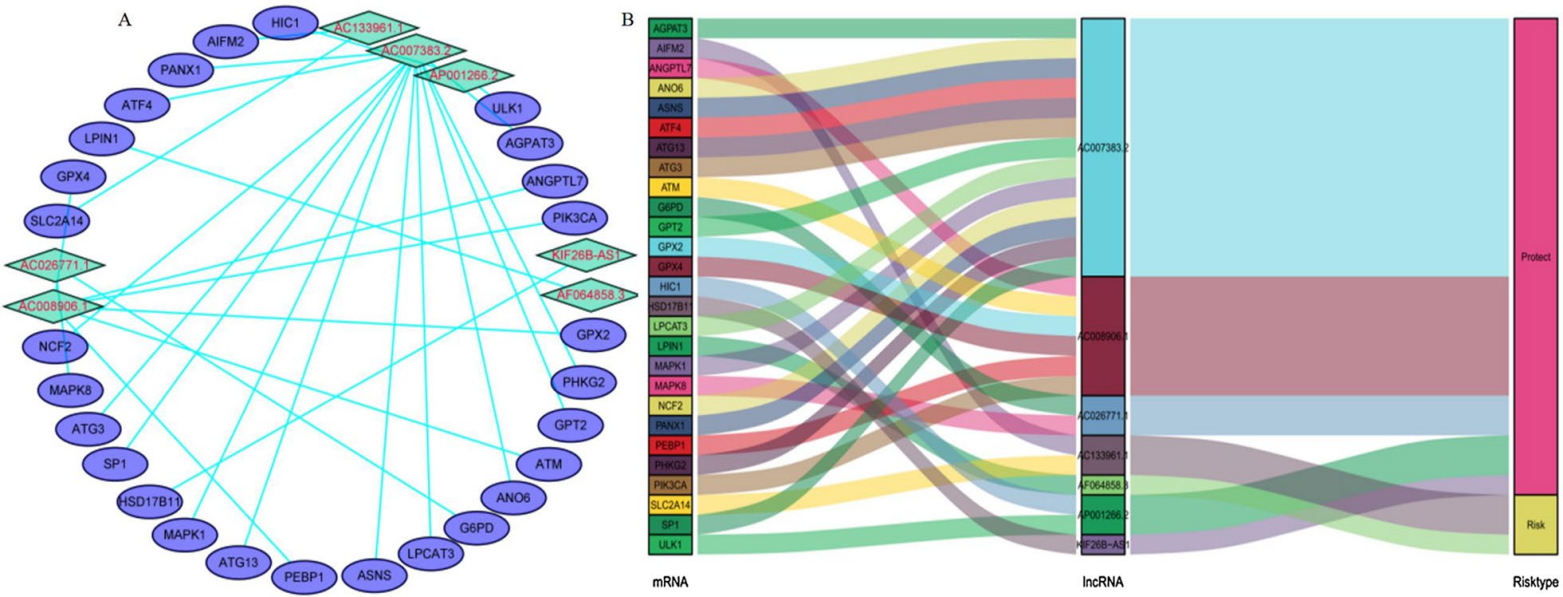
Cytoscape and Sankey diagram of long non-coding RNA (lncRNA)-mRNA co-expression network

**Fig. 8:**
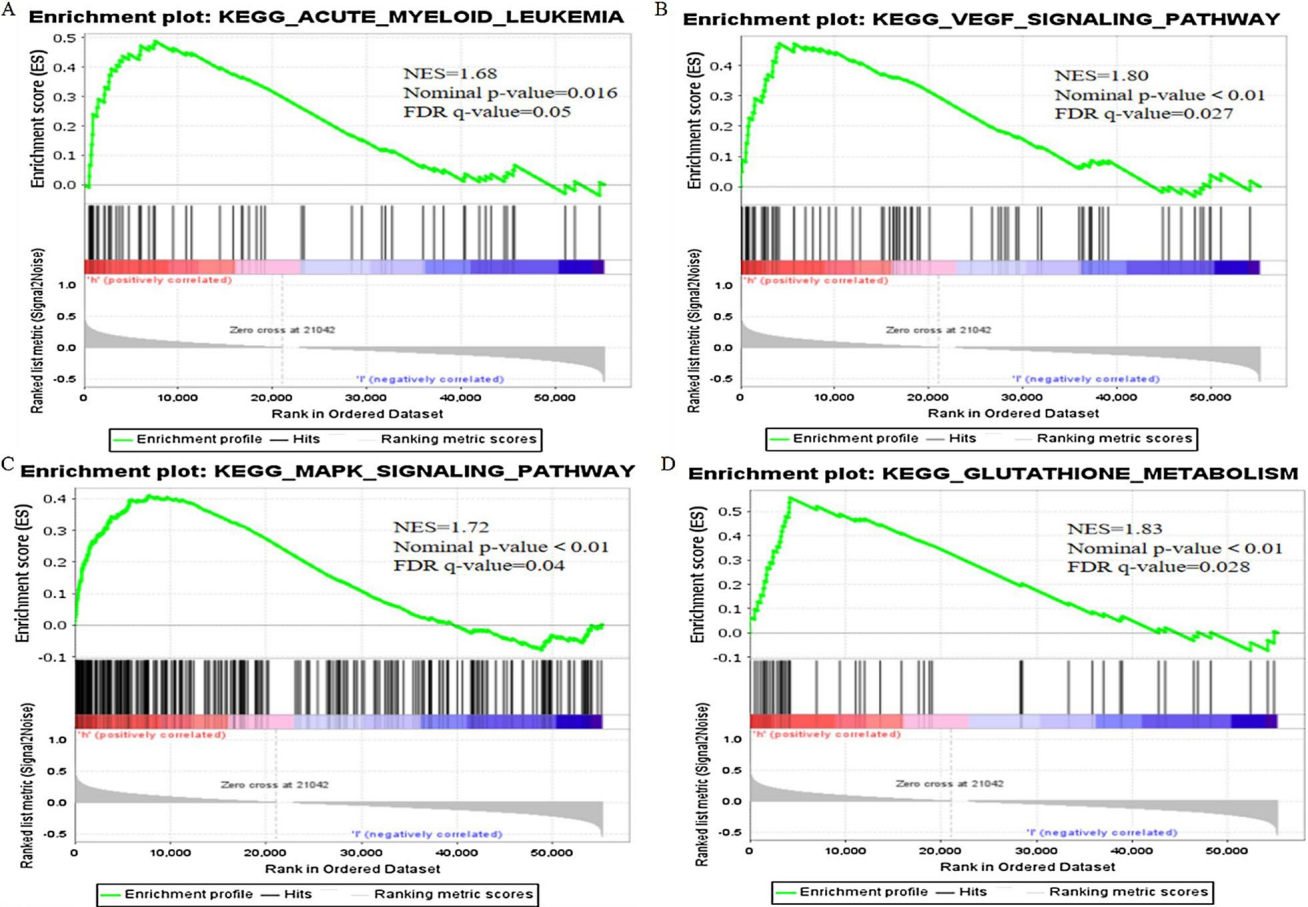
Gene set enrichment analysis (GSEA). **a** Acute myeloid leukemia (AML), **b** vascular endothelial growth factor (VEGF), **c** mitogen-activated protein kinase (MAPK) signaling pathways, and **d** glutathione metabolism

**Fig. 9:**
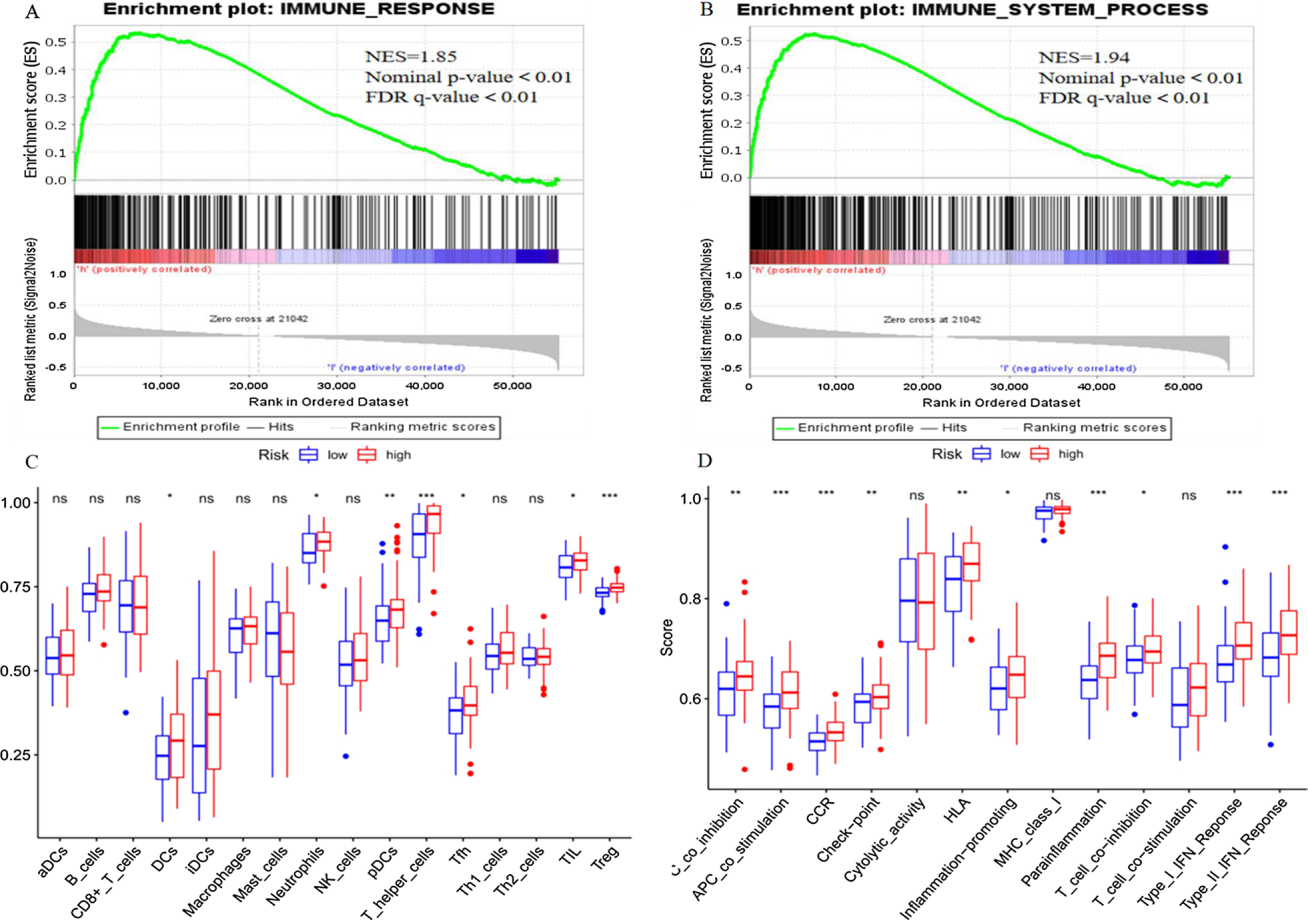
Correlations between long non-coding RNA (lncRNA) signatures and immune cell infiltration in patients with acute myeloid leukemia (AML). **a** Immune response. **b** Immune system process. **c** Single-sample gene set enrichment analysis (ssGSEA) scores of 16 immune cells and **d** 13 immune-related functions in high- and low-risk groups. aDCs, activeted dendritic cells; DCs, dendritic cells; iDCs, immature DCs; pDCs, plasmacytoid dendritic cells; Tfh, Follicular helper T cell; TIL, tumor infiltrating lymphocyte; Tregs, regulatory T cells

Cytoscape software was used to visualize the co-expression network between lncRNAs and mRNAs (Fig. 7a). We also further used the "ggalluvial" package in R to draw a Sankey diagram to determine whether lncRNAs are protective or risk factors (Fig. 7b). Next, to further explore the potential biological function of seven ferroptosis-related lncRNAs, we performed a GSEA of the high- and low-risk groups. The results showed that several tumor-related pathways were enriched in the high-risk group, such as the AML (Fig. 8a), vascular endothelial growth factor (VEGF, Fig. 8b), and mitogen-activated protein kinase (MAPK, Fig. 8c) signaling pathways. Moreover, the high-risk group was enriched in ferroptosis-related biological pathways, including that for glutathione metabolism (Fig. 8d). In addition, the results of the immune-related GSEA enrichment analysis showed that the immune response (Fig. 9a) and immune system processes (Fig. 9b) in the high-risk group were significantly enriched.

### Evaluation of relationship between risk score and immune infiltration

To further analyze the immune cell infiltration of patients with AML and explore the differences in immune status between high-risk and low-risk populations, we used ssGSEA to estimate the difference in the enrichment scores of immune cells and immune-related functional pathways in the high- and low-risk groups. The results showed that the immune cell subpopulations of dendritic cells (DCs), neutrophils, plasmacytoid DCs (pDCs), T helper cells, T follicular helper cells, tumor-infiltrating lymphocytes, and regulatory T cells (Tregs) were significantly upregulated in the high-risk group (all *P* < 0.05, Fig. 9c). APC co-inhibition, APC co-stimulation, chemokine receptors (CCR), Check-point, human leukocyte antigen, inflammation-promoting, para-inflammation, T-cell co-inhibition, and type I and II interferon responses were significantly increased in the high-risk group (all *P* < 0.05, Fig. 9d).

## Discussion

Although some progress has been made in the screening, diagnosis, and treatment of AML in recent years, it is still one of the most fatal malignant hematological tumors because of its complex underlying genetic and molecular mechanisms of development [25]. Ferroptosis, a newly defined regulatory cell death, is caused by the excessive accumulation of lipid peroxides and iron-dependent reactive oxygen species [26]. Recent studies have shown that ferroptosis is closely related to the pathophysiological processes of many diseases, including AML [27]. As an emerging gene and molecular biomarker, IncRNA plays an important role in various biological processes in AML [28]. However, the research on the relationship between ferroptosis-related lncRNAs and the prognosis of cancer patients is still in its infancy. Through bioinformatics analysis, Tang et al. found that ferroptosis-related lncRNAs can be used to predict the prognosis of head and neck squamous cell carcinoma [29]. Regrettably, there is still a lack of studies on ferroptosis-related lncRNAs to predict the prognosis of patients with AML. Therefore, in this study, we attempted to construct a prognostic model for patients with AML by exploring ferroptosis-related lncRNAs to improve their survival rate.

In our study, we first obtained 259 ferroptosis-related genes and then 2977 ferroptosis-related lncRNAs using correlation analysis. Patients with AML were randomized into the training and validation groups at a ratio of 7:3. In the training group, 344 prognostic ferroptosis-related lncRNAs were identified using univariate Cox regression. To avoid overfitting, we used a LASSO regression for dimensionality reduction to obtain 13 ferroptosis-related lncRNAs. Finally, we used multivariate Cox regression analysis to construct a signature containing the 7 ferroptosis-related lncRNAs with the lowest AIC value. Based on the risk score, patients were divided into high- and low-risk groups. The results of univariate and multivariate Cox regression analyses of the training and validation groups both indicated that the risk score was an independent risk factor affecting the prognosis of patients with AML. The AUC, PCA, and T-SNE further confirmed the accuracy and distinguishing ability of the lncRNA signature.

The Sankey diagram intuitively showed that AC133961.1 and AF064858.3 were risk factors for the prognosis of patients with AML and AP001266.2, AC007383.2, AC008906.1, AC026771.1, and KIF26B-AS1 were identified as prognostic protective factors. Our findings indicated that high expression levels of AC133961.1 and AF064858.3 were associated with poor risks categorized using cytogenetics, which may be key risk factors for the prognosis of patients with AML.

To explore the biological function of the seven ferroptosis-related-lncRNA signature for the prognosis of AML, the GSEA method was used to compare the low- and high-risk groups. The AML, VEGF, and MAPK signaling pathways were significantly enriched in the high-risk group. Previous studies have confirmed that VEGF and MAPK signaling pathways are involved in the regulation of ferroptosis in various diseases [30, 31]. In addition, the glutathione metabolism pathway was significantly enriched in the high-risk group. Glutathione plays an important role in the redox reaction of the body, and inhibition of its synthesis is a key step in ferroptosis [32, 33].

Moreover, the GSEA showed that immune response and immune system processes were significantly enriched in the high-risk group. Presently, immunotherapy is an important intervention for leukemia [34], and the study of immune infiltration is essential to clarify the relationship between tumor and immunity. Therefore, we used the ssGSEA algorithm to study immune cell infiltration and immune function activation in patients with leukemia. We noticed that the high-risk group exhibited higher expression levels of Tregs, pDCs, and neutrophils. Treg-mediated immunosuppression is an important mechanism of tumor immune evasion [35]. An increase in the number of pDCs promotes human immune escape and is associated with poor prognosis [36]. Moreover, neutrophils secrete VEGF and inhibit T cell response activity to promote tumor growth and metastasis [37, 38]. In addition, CCRs were enriched in the high-risk group. The combination of the CCR and its ligand promotes the recruitment of Tregs and myeloid-derived suppressor cells to tumor lesions, which promotes tumor growth and metastasis [39, 40]. Recent research advances are gradually elucidating the unclear and complicated relationship between immunity and ferroptosis [41]. Wang et al. [42] found that immunotherapy-activated CD8 + T cells enhance ferroptosis-specific lipid peroxidation in tumor cells, and that increased ferroptosis contributes to the antitumor efficacy of immunotherapy. In our study, we found that patients in the high-risk group had a large number of immunosuppressive cell infiltrations and high expression of anti-tumor immune function. We speculate that there may be a correlation between immune escape and anti-leukemia cell ferroptosis, which leads to a poor prognosis for patients, and additional studies are warranted to confirm these results.

Recent breakthroughs in ferroptosis and immunity research have provided potential new treatment strategies for diseases. Furthermore, novel and useful functions of lncRNAs are constantly being discovered. However, there are still many gaps in the understanding of the relationships between ferroptosis and immunity. Therefore, we identified seven ferroptosis-related lncRNAs using a high-throughput sequencing technology and further elucidated their relationship with immunity. However, this study had some limitations. First, we only used test and validation groups in the TCGA database to verify the effectiveness of the lncRNA prognostic model related to ferroptosis. Second, we did not conduct basic experiments and clinical samples to verify the expression level and function of the identified ferroptosis-related lncRNAs, so our results reliability cannot be fully guaranteed. Similarly, our research results should be used with caution. Despite its limitations, to our knowledge, this is the first study to construct a ferroptosis-related lncRNA prognostic signature in AML. Furthermore, our future research will focus on these ferroptosis-related lncRNAs to investigate strategies for providing a new treatment method for patients with AML.

## Conclusion

In conclusion, we identified and verified a seven ferroptosis-related lncRNA signature (AP001266.2, AC133961.1, AF064858.3, AC007383.2, AC008906.1, AC026771.1, and KIF26B-AS1) with independent prognostic value in patients with AML. Therefore, we propose that this lncRNA signature could be a potentially useful prognostic indicator in the future and inspire new ferroptosis treatment methods to improve the prognosis of patients with AML.

## Supplementary Information

**Additional file 1.** The annotation of 16 immune cells and 13 functions in single-sample gene set enrichment analysis (ssGSEA).

**Additional file 2.** 259 Ferroptosis-related genes.

**Additional file 3.** 344 Ferroptosis-related long non-coding RNAs (lncRNAs).

**Additional file 4. Figure 1**: Correlation between the expression level of 7 ferroptosis-related long non-coding RNAs and risk scores in the entire corhort. (A–G) AP001266.2, AC133961.1, AF064858.3, AC007383.2, AC008906.1, AC026771.1, and KIF26B-AS1, respectively. NS: not significant; *P < 0.05; **P < 0.01; ***P < 0.001.

**Additional file 5.** TCGA-LAML accession numbers.

## Data Availability

The raw data of this study are derived from the TCGA database (https://portal.gdc.cancer.gov/), the specific sample is TCGA-LMAL, and the detailed accession number is shown in Additional file 5, which are publicly available databases.
