# Supplementary figures and images for: Identification of seven novel ferroptosis-related long non-coding RNA signatures as a diagnostic biomarker for acute myeloid leukemia
Source: BMC Med Genomics. 2021 Sep 27;14:236. doi: 10.1186/s12920-021-01085-9 (PMC8474743; doi:10.1186/s12920-021-01085-9)

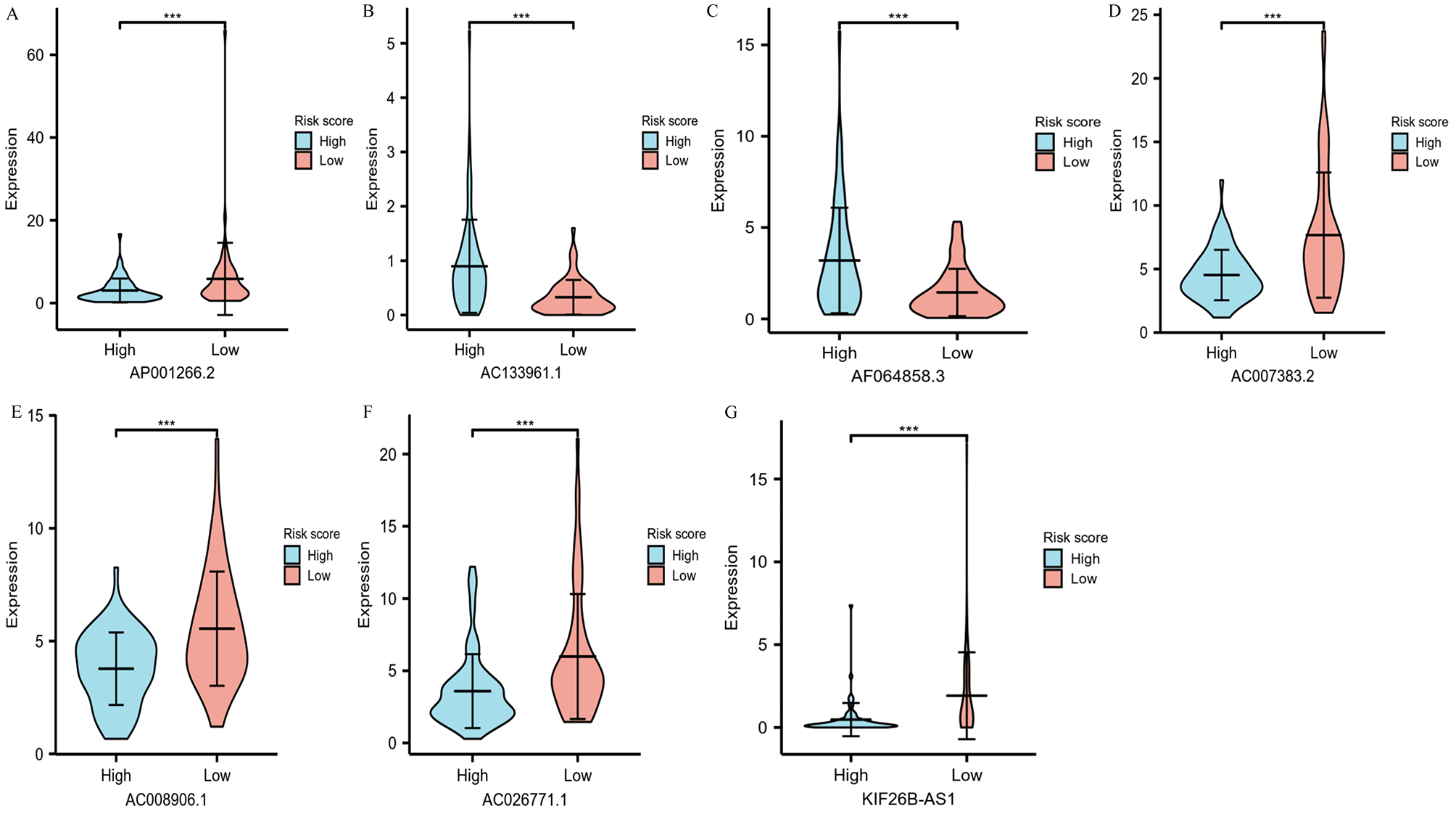

Supplement: Supplementary file 4 — Additional file 4. Figure 1: Correlation between the expression level of 7 ferroptosis-related long non-coding RNAs and risk scores in the entire corhort. (A–G) AP001266.2, AC133961.1, AF064858.3, AC007383.2, AC008906.1, AC026771.1, and KIF26B-AS1, respectively. NS: not significant; *P < 0.05; **P < 0.01; ***P < 0.001. [file 12920_2021_1085_MOESM4_ESM.png]
